# Proceedings of the fourth international conference on central hypoventilation

**DOI:** 10.1186/s13023-014-0194-5

**Published:** 2014-12-05

**Authors:** Ha Trang, Jean-François Brunet, Hermann Rohrer, Jorge Gallego, Jeanne Amiel, Tiziana Bachetti, Kenneth H Fischbeck, Thomas Similowski, Christian Straus, Isabella Ceccherini, Debra E Weese-Mayer, Matthias Frerick, Katarzyna Bieganowska, Linda Middleton, Francesco Morandi, Giancarlo Ottonello, the European Central Hypoventilation Syndrome Consortium

**Affiliations:** French Centre of Reference for Central Hypoventilation, Robert Debré University Hospital, EA 7334 REMES Paris-Diderot University, 48 boulevard Serurier, 75019 Paris, France; IBENS, CNRS 8197, INSERM 1024, École normale supérieure, Paris, France; Research Group Developmental Neurobiology, Department of Neurochemistry, Max Planck Institute for Brain Research, Frankfurt am Main, Germany; Inserm U676, Robert Debré University Hospital, Paris, France; French Centre of Reference for Central Hypoventilation, Necker-Enfants Malades University Hospital, Paris, France; Istituto Giannina Gaslini, Genova, Italy; Neurogenetics Branch, National Institute of Neurological Disorders and Stroke, National Institutes of Health, Bethesda, Missouri USA; French Centre of Reference for Central Hypoventilation, La Pitié Salpêtrière University Hospital, Pierre et Maris Curie University, Paris, France; Laboratorio di Genetica Molecolare, Istituto Giannina Gaslini, Genova, Italy; Autonomic Medicine in Paediatrics (CAMP), Ann & Robert H. Lurie Children’s Hospital of Chicago, Northwestern University Feinberg School of Medicine, Chicago, Illinois USA; Klinikum Dritter Orden, Munich, Germany; Children’s Memorial Health Institute in Warsaw, Warsaw, Poland; Parent and UK CCHS Support Network, Oxford, UK; Ospedale Sacra Famiglia, Erba, Italy; Via Gerolamo Gaslini, Genova, Italy

**Keywords:** Central hypoventilation, Autonomic nervous system, Congenital central hypoventilation syndrome, *PHOX2B* gene, Hirschsprung’s disease, Alanine expansion, Central control of breathing, Home mechanical ventilation, Phrenic nerve stimulation, Diaphragmatic stimulation

## Abstract

Central hypoventilation syndromes (CHS) are rare diseases of central autonomic respiratory control associated with autonomous nervous dysfunction. Severe central hypoventilation is the hallmark and the most life-threatening feature. CHS is a group of not-fully defined disorders. Congenital CHS (CCHS) (ORPHA661) is clinically and genetically well-characterized, with the disease-causing gene identified in 2003. CCHS presents at birth in most cases, and associated with Hirschsprung’s disease (ORPHA99803) and neural crest tumours in 20% and 5% of cases, respectively. The incidence of CCHS is estimated to be 1 of 200,000 live births in France, yet remains unknown for the rest of the world. In contrast, late-onset CHS includes a group of not yet fully delineated diseases. Overlap with CCHS is likely, as a subset of patients harbours PHOX2B mutations. Another subset of patients present with associated hypothalamic dysfunction. The number of these patients is unknown (less than 60 cases reported worldwide). Treatment of CHS is palliative using advanced techniques of ventilation support during lifetime. Research is ongoing to better understand physiopathological mechanisms and identify potential treatment pathways.

The Fourth International Conference on Central Hypoventilation was organised in Warsaw, Poland, April 13–15, 2012, under the patronage of the European Agency for Health and Consumers and Public Health European Agency of European Community. The conference provided a state-of-the-art update of knowledge on all the genetic, molecular, cellular, and clinical aspects of these rare diseases.

## Introduction

The Fourth International Conference on Primary Central Hypoventilation, SCIENCE & LIFE OF CHS, was held in Warsaw, Poland, April 13–15, 2012. The meeting was under the patronage of the European Agency for Health and Consumers (EAHC) and Public Health European Agency (PHEA) of European Community. It was organised by the European CHS Consortium.

The conference aimed to provide an update of knowledge recently acquired on : i) functional, molecular and genetic aspects of central control of breathing, of Autonomic Nervous System Dysfunction and of Central Hypoventilation Syndromes (CHS) (ORPHA661); ii) on epidemiology, diagnosis, ongoing care management, health care standards of and service organization for CHS. The conference also provided an understanding of the current state of CHS care across the world and an opportunity to exchange ideas to improve service and care organisation in the future.

### What we know and what we don’t know: The unknowns are yet to surface

***Ha TRANG,****MD, PhD. French Centre of Reference for Central Hypoventilation, Robert Debré University Hospital, EA 7334 REMES, Paris-Diderot University, Paris, France*

Central hypoventilation syndromes (CHS) are rare disorders of central autonomic respiratory control and global dysfunction of the autonomic nervous system. These result in alveolar hypoventilation during spontaneous breathing or ventilatory dependency with the absence or reduction of a response to CO_2_. CHS include a heterogeneous group of not fully characterized diseases. Congenital CHS (CCHS) is a clinically and genetically well-characterized disease. In contrast, late-onset CHS includes a group of not yet fully delineated diseases. Overlap with CCHS is likely, as a subset of patients harbours Paired-like homeobox 2b *(PHOX2B)* mutations. Another subset of patients present with associated hypothalamic dysfunction. Ventilation support using advanced techniques is required for life. Large disparities exist in access to diagnosis and treatment across Europe. The long-term outcomes are unknown.

Respiration and the respiratory system are under two main control systems: the cortex which provides voluntary or behavioural control, and the bulbo-pontine in the brain stem which provides autonomic or chemical control.

In his very first hours of life, a baby with CCHS becomes cyanotic with severe desaturation. He breathes very slowly and superficially, may present with apnoeas, but makes no effort to breathe. Rapidly, intubation and ventilation are required. Polysomnographic recordings show slow and shallow breathing, with a reduction in the respiratory rate and the amplitude of ventilation. This results in alveolar hypoventilation, desaturation and hypercapnia. These features are more marked in sleep than in wakefulness and during non-Rapid Eye Movement (non-REM) sleep than in REM sleep [1-3]. As a matter of fact, non-REM sleep is a stage in sleep which is almost entirely under chemical control. Nevertheless, the absence or reduction of ventilatory response to CO_2_ is present, whatever the state of alertness.

In 2003, the discovery of *PHOX2B* mutations in CCHS patients was a major step forward in our understanding of the disease [4,5]. By extending our knowledge through basic research, molecular, cellular, and animal studies, it is now known that the *PHOX2B* gene controls neuron differentiation very early during pregnancy, and the brainstem retro-trapezoid nucleus (RTN) in which *PHOX2B* is expressed is found to be crucial in control of breathing [6,7].

However, does *PHOX2B* mutation wholly account for the manifestation of CCHS? Are RTN cells the only neuronal cells underlying these respiratory deficits? Investigations using new-born mice carrying the *PHOX2B* mutation are providing new insights into neuronal involvement in CCHS [8,9]. The mutant animals have a similar phenotype to humans and present with apnoeas and reduced hypercapnic ventilatory responses.

CCHS is associated with major conditions such as Hirschsprung’s disease and neural crest tumours [10-12]. Is there a genetic link underlying these conditions? During the conference, outstanding experts will highlight on how *PHOX2B* may interact with the genetic codes from these diseases, to produce the major CCHS phenotypes. An update will be made on *PHOX2B* anomalies, sessions on inheritance and penetrance, genetic counselling and future potential treatments.

Diagnosis and management of CCHS remains problematic, especially as new presentations of the condition emerge. Milder forms of disease have been observed in babies who at birth present with apnoeas and desaturation, only to improve within weeks and in some cases do not require ventilatory support. On genetic testing, these individuals are shown to have a +5 alanine expansion, and on monitoring sleep parameters have all the features of CCHS: sleep desaturation, and increased tidal CO_2_ to 59 mmHg max, averaging around 54–55 mmHg. Genetic studies of a family revealed that the mother had a +5 alanine expansion, but no clinical symptoms, and the father had no *PHOX2B* abnormalities. These new presentations raise diagnostic and management issues and questions relating to the categorisation of the disease.

There are many more management issues to confront us including the appropriate mode of ventilation support for patients, and whether specific criteria apply to cardiac pacing in CCHS patients compared with the general population. Can follow-up and care be tailored to each patient? What are the best clinical pathways for transition from paediatric to adult care?

At present, there is a paucity of information on the location of CHS patients, the mortality rate and the incidence of CCHS, currently estimated at one in 200 000 births [13]. It is most likely that the incidence of CCHS is much higher. Also, other questions need to be addressed such as, where the experts in CHS are, how CHS is diagnosed or managed in each country, and what guidelines for diagnosis, treatment, and for carers are used. A consensus is required on information for families and carers.

To address these issues, the European CHS Project was initiated by the EUCHS Consortium. It is co-financed by the EAHC, an official agency of the European Community, and six national public institutions from France, Germany, Italy, Poland, Sweden and United Kingdom. The project includes altogether 11 European countries (Spain, Portugal, Austria, Slovenia, and Croatia) that account for 70% of EU population. This is a three-year project with a 6-month extension. The objective of the project is to develop a secure web-based CHS registry to describe the demographics of the condition, medical practice in Europe, and serve as a database for research and health policy decisions. In addition, a guideline for diagnosis and treatment of CHS, and a multilingual patient and carer information booklet will be produced. Expert centres will be identified, existing service for CHS in Europe will be evaluated and a multilingual website set up to facilitate communication between patients and healthcare professionals.

The secure web CHS registry is the first site to gather population-based data and has been operating for a year. Emerging data is providing an insight into the expert centres in Europe which can now be identified on an interactive map on the web site. The European guidelines, developed by EUCHS for CHS, are targeted at healthcare professionals and the first draft is currently under review, before publication. The patient and carer information booklet, in multi-lingual formats, are available on the website. This 4^th^ International Conference on Central Hypoventilation is organised as part of the EUCHS Project to gather and exchange knowledge between healthcare professionals, families, researchers and clinicians.

### Neuronal lesions underlying the respiratory defects of CCHS

***Jean-François BRUNET****, PhD. IBENS, CNRS 8197, INSERM 1024, École normale supérieure, Paris, France*

CCHS is a complex dysautonomia with variable genetic penetrance [12]. The majority of CCHS symptoms and associated conditions, such as neuroblastoma and Hirschsprung’s disease, arise from the dysfunction of the visceral nervous system [11,12]. For this reason, the pan-visceral homeobox transcription factor *PHOX2B* was originally tested as a candidate gene and later confirmed as the causative gene in CCHS [4,5].

*PHOX2B* is expressed in a limited set of neuronal circuits during embryonic development and throughout adult life. These circuits are all related in that they regulate vital functions: cardiovascular, digestive and respiratory. The majority of *PHOX2B* neurons are in the motor and sensory branches of these circuits. In the sensory pathway, *PHOX2B* is expressed in three cranial sensory ganglia: geniculate, petrosal, nodose, which monitor vital parameters such as blood pressure, the chemical composition of the blood and digestive contents. It is also expressed in the nucleus of the solitary tract (nTS) where this information is integrated and fed back continuously to the *PHOX2B*-expressing autonomic ganglia (parasympathetic, sympathetic and enteric), through their preganglionic neurons in the central nervous system [14]. In *PHOX2b* knockout mice, all these neurons are absent, indicating that *PHOX2B* is essential for the development and function of nerve cells within this system.

The defining symptom of CCHS is respiratory: hypoventilation with apneic episodes, usually during sleep. The disorder ranges from potentially lethal respiratory distress at birth to late-onset respiratory arrests, and is accompanied by the absence of hyperventilation in response to hypercapnic challenge [10].

In the circuits that control breathing, *PHOX2B* is expressed and required in the nodose ganglion which contains the neurons responsible for the pulmonary stretch or Herring-Breuer reflex, in the carotid body (CB) which contains oxygen and CO_2_ sensors, in the petrosal ganglion that innervates the CB, and in the nTS that integrates all this mechano-, chemo- and barosensory information. *PHOX2B* is also expressed and required in several noradrenergic centres, such as the locus ceruleus and A5, which modulate respiratory rhythm. It is also a determinant of branchial motor neurons (for example in the facial motor nucleus and the nucleus ambiguous), which are the respiratory motoneurons in the aquatic ancestors of air-breathing vertebrates (including humans), in whom they have kept a minor role in breathing. Thus, the known sites of action of *PHOX2B*, while revealing a strong implication in breathing, do not readily explain the respiratory symptoms of CCHS, and suggest the involvement of other neurons. As it turns out, *PHOX2B* is also expressed in interneurons in the hindbrain whose function is unknown and might explain these respiratory symptoms.

To model CCHS in animals, one of the most common CCHS-causing mutations, a +7 alanine expansion of the *PHOX2B* gene, was introduced into mice. Mice harboring this mutation died at birth of respiratory failure [15]. Plethysmographic analysis performed prior to death revealed an absence of chemoreflex response to hypercapnia. Histological analysis showed a massive loss of *PHOX2B*-expressing, glutamatergic (Vglut2^+^), neurokinin-1 receptor-positive neurons that form the RTN, located at the surface of the ventrolateral medulla below the facial motor nucleus.

In anaesthetized rats, RTN was previously shown to be sensitive to blood levels of CO_2_ [16]. Electrophysiological data also showed that RTN *PHOX2B*^+^ neurons establish excitatory glutamatergic synapses with the pre-Bötzinger complex, the respiratory pacemaker, to accelerate respiratory rhythm at birth. If the RTN is substantially deficient or absent, as in knock-in mice carrying a +7 alanine expansion of the *PHOX2B* gene, the animals die at birth from respiratory failure.

RTN *Phox2b*^+^ neurons express the basic-helix-loop-helix (bHLH) transcription factor, “atonal homolog 1” (Atoh1) (also expressed around embryonic day 12.5 in proliferating rhombic lip progenitors with no role in respiration described to date). Tracking of post-mitotic neurons of the RTN that express Atoh1 revealed that they migrate with the facial nucleus (but do not require its presence to do so). The expression of *Atoh1* in the RTN region is abolished in mice carrying a +7 alanine expansion mutation in *PHOX2B*. This marker thus allows us to follow the fate of RTN neurons conditional *PHOX2B* mutations, as exemplified below.

The *Phox2b*^*+*^*/Atoh1*^*+*^ neurons of the RTN arise from embryonic neuronal precursors with a history of expression of two other transcription factors: Early Growth Response Protein 2 (Egr2) and Ladybird homeobox 1 (Lbx1), as demonstrated by genetic tracing studies in mice, using either a *Lbx1::Cre*, or a *Egr2::Cre* allele, combined with a *β-galactosidase* reporter gene. Crossing the *Lbx1::Cre* or the *Egr2::Cre* allele with a floxed *PHOX2B* allele (i.e. conditional null allele) to produce *Phox2b*^*lox/lox*^*; Egr2*^*cre/+*^ or *Phox2b*^*lox/lox*^*;Lbx1*^*cre/+*^ genetic backgrounds, thus allows to destroy the RTN while affecting much fewer neurons than in *Phox2b*^*27ala*^ mutants (where the deleterious mutation is expressed in all *Phox2b*^*+*^ neurons) .

In vitro experiments have compared the rhythmic, respiratory-like activity of the hindbrain-spinal cord from mice embryos at E14 in wild type and *PHOX2B* mutant backgrounds (*Phox2b*^*27ala*^, *Phox2b*^*lox/lox*^; *Egr2*^*cre/+*^ and Phox2b ^lox/lox^; Lbx1 ^cre/+^). Optical recordings of calcium uptake (a measure of rhythmic activity) at pH 7.4 and pH 7.2 (a proxy for hypercapnic challenge) showed a rhythmic activity in the RTN region at pH 7.4 and an acceleration of this activity at pH 7.2 in control animals. In contrast, there was no rhythmic activity at either pH in the RTN region of +7 alanine *PHOX2B* expansion and conditional *PHOX2B* mutants, showing the functional absence of a RTN. This finding was confirmed in electrophysiological recordings of the phrenic nerve at E16, where rhythmic activity is normally accelerated at an acidic pH, but not in the +7 alanine *PHOX2B* mutant or the conditional knockouts, showing that entrainment by the CO_2_-sensitive RTN of the respiratory pacemaker (the pre-Bötzinger complex) does not occur [17].

A mutation even more selective for the RTN was then engineered, which consists in pairing a conditional +7 alanine *PHOX2B* allele with the *Egr::Cre* or a *Lbx1::Cre* allele: this combination most likely spares many neurons affected in the conditional nulls (*Phox2b*^*lox/lox*^; *Egr2*^*cre/+*^ or *Phox2b*^*lox/lox*^*; Lbx1*^*cre/+*^ mutants), since the majority of neurons that depend on *PHOX2B* are not sensitive to the +7 alanine mutation. In these mutant backgrounds, the RTN was histologically and functionally abolished, as in the previous *PHOX2B* mutants analyzed, strengthening the case for the RTN as the chemoreflex center. However, unlike the conditional nulls, the animals survived [8].

This unexpected outcome shows that the central chemoreflex, long considered as the paramount drive to breathe, is not required in mice for survival. It suggests that there are one or more other *Phox2b*-positive (and *Phox2b*^*27ala*^-sensitive) populations of neurons which, either by themselves or in combination with the deletion of the RTN and consequent lack of chemoreflex, cause respiratory arrest at birth, in mice and possibly also in humans. Our focus is now on the quest for this population of neurons that, in combination with the RTN, might explain the full respiratory phenotype of CCHS.

### PHOX2B and midkine/Alk signalling in the control of sympathetic neuron proliferation and neuroblastoma predisposition

***Hermann ROHRER****. Research Group Developmental Neurobiology, Department of Neurochemistry, Max Planck Institute for Brain Research, Frankfurt am Main, Germany*

Development neurobiologists study the generation of neurons from progenitors and the mechanisms that control their specification and differentiation. This approach has been used to follow the fate of specific groups of neurons in the autonomic nervous system and to investigate the molecular mechanisms that predispose neuroblasts to develop neuroblastoma, a tumour arising in sympathetic ganglia and the adrenal medulla.

Neurons and glial cells of the peripheral nervous system differentiate from precursor cells of the neural crest. Neuronal development is controlled by a network of transcription factors including *PHOX2B*, *ASCL1*, *GATA3* and *HAND2* [18]. They control distinct cell functions, such as differentiation, migration, proliferation and survival in both the embryonic and adult peripheral nervous system.

During neurogenesis, undifferentiated precursor cells divide asymmetrically to produce both a precursor cell that remains undifferentiated and a neuron progenitor cell that differentiates to a post-mitotic neuron. In contrast to this canonical pattern of neurogenesis found throughout the nervous system, in sympathetic ganglia, neuronal cells continue to divide after acquisition of neuronal properties. Differentiation of sympathetic neuron precursors is, therefore, not linked to cell cycle withdrawal. This raises the suspicion that neurogenesis is less well controlled in the sympathetic lineage compared to other cells of the autonomic nervous system, and may lead to tumours, such as, neuroblastoma [19].

Neuroblastoma arises from developing embryonic sympathetic neurons, and is the most frequent extra-cranial solid tumour in children, accounting for 10 per cent of all childhood tumours. It has a broad clinical spectrum ranging from benign tumours that spontaneously regress to aggressive tumours that metastasize to the bone. Around 40% of tumours are aggressive, for which there is no treatment. Mutations in *PHOX2B* gene and anaplastic lymphoma kinase (Alk) have been identified in both familial and sporadic cases of the neuroblastoma [20].

The proliferation and differentiation of immature neuroblasts has been studied in response to wild type and mutant PHOX2B proteins ectopically expressed in cultures of chick and embryonic sympathetic neurons. *PHOX2B* variants with point mutations in the homeodomain were used. The *PHOX2B* wild type gene had strong anti-proliferative effects in sympathetic neuroblasts, which is lost in mutant variants. The *PHOX2B* wild type may therefore act as a tumour suppressor gene which is de-repressed by *PHOX2B* mutations in the homeodomain. Alternatively, the mutant proteins may affect a gain-of-function. To test this hypothesis, the experiment was repeated after knockdown of endogenous *PHOX2B* using siRNA, to mimic the situation of a heterozygous *PHOX2B* mutation in neuroblastoma. Under these conditions, *PHOX2B* variants with mutations in the homeodomain increase the proliferation of immature sympathetic neurons. The increased proliferation is blocked by HAND2 knockdown and restored by over expression of HAND2. HAND2 was precipitated from sympathetic neuron not only with the wild type but also with the *PHOX2B* mutant variants. Complexes of HAND2/wild type PHOX2B appear to inhibit proliferation, while complexes of HAND2/mutant PHOX2B have a proliferative effect on immature sympathetic neurones [21].

Neuroblastoma predisposition may be caused by a deleterious gain-of-function which results in increased proliferation of sympathetic neurons, since sympathetic neural proliferation is controlled by the interaction of *HAND2* and *PHOX2B*. The differential effects of *PHOX2B* mutations in the sympathetic and parasympathetic ganglia may, in part, be explained by different regulatory mechanisms and, specifically the role of *HAND2. PHOX2B* mutation variants have different functional consequences depending interaction with other mediators, expression levels or post-inscriptional modifications. *PHOX2B* when highly expressed may either affect proliferation during neural development or, may cause the cell’s exit from the mitotic cycle. Similar conclusions have been reached from studies of the transcription factor, STL1, which binds not only to promoters of neural differentiation but also to promoters controlling cell proliferation.

Alk is a trans-membrane receptor tyrosine kinase and predisposing Alk germline mutations are found in familiar forms of neuroblastoma, and also in 8% of sporadic neuroblastoma. The effect of Alk mutations have been investigated in immature chick sympathetic neurons. Forced expression of wild-type Alk and neuroblastoma-related constitutively active Alk mutants in cultures of proliferating immature sympathetic neurons increased proliferation of these cells, whereas pharmacological Alk inhibitors, overexpressed Alk and, knockout Alk mutations decreased proliferation [22].

Midkine, which is highly expressed in sympathetic ganglion has been identified as a ligand for the Alk receptor. In cultures of proliferating immature chick sympathetic neurons, Midkine-Alk signalling is essential and sufficient to stimulate sympathetic neuron proliferation. In vivo inhibition of Alk-midkine signalling by virus-mediated siRNA knockout of Alk and midkine leads to the formation of smaller sympathetic ganglia and reduced proliferation of both tyrosine hydroxylase- and *PHOX2B*-positive cells, confirming the role of midkine-Alk signalling in sympathetic neuron proliferation. Stimulation of midkine-Alk receptor signalling increases the expression of nMyc and trkB - two genes which are associated with aggressive forms of neuroblastoma [22].

Midkine-Alk signalling is specific to sympathetic ganglia. It regulates HAND2 mediated cell proliferation and ganglion size by an auto regulatory mechanism. Increased Nmyc expression may cause elevated and sustained Alk-signalling leading to a predisposition to neuroblastoma [22]. Both PHOX2B and Alk mutations may increase and prolong proliferation of embryonic sympathetic neurones in mouse models, increasing the probability of additional mutations within this cell lineage, leading to the clinical manifestation of neuroblastoma. Attempts to prove this hypothesis are currently being undertaken.

### CCHS: lessons from newborn mice

***Jorge GALLEGO,****PhD. Inserm U676, Robert Debré University Hospital, Paris, France*

Mice are the preferred animal model for genetic studies. As CCHS occurs in newborns, studies were undertaken in neonatal mice. Three mutant mice models, were developed which survived the early postnatal period: knock-out mice with one invalidated allele of *PHOX2B* (*PHOX2B*^+/−^) which allowed the study of postnatal respiratory breathing and related functions such as thermoregulation; a constitutional +7 alanine *PHOX2B* expansion mutation (PHOX2B^27Ala^) that die within a few hours following birth and, a conditional mutant *PHOX2B*^27Ala^ in which the mutation was confined to the RTN.

In all these studies, breathing variables, heart rate, body temperature and markers of behavioural activity were measured non-invasively in neonatal mutant mice, by whole-body flow barometric plethysmography, under baseline conditions and, during exposure to chemical (hypoxia and hypercapnia) and thermal stimuli. All parameters were measured in mice during quiet sleep.

#### *PHOX2b* knockout mice

Null *PHOX2b*^*−/−*^ mice die in utero but mice carrying one invalidated P*HOX2b* allele (*PHOX2b*^*+/−*^) survive and are fertile, and thus do not reproduce the CCHS phenotype [9]. The ventilatory response to hypercapnia in unrestrained *PHOX2b*^*+/−*^ newborn mice was about 40% smaller in PHOX2b^+/−^ than PHOX2b^+/+^ pups. CO_2_-sensitive sites such as the locus coeruleus and area postrema, the RTN, and afferent pathways from the carotid bodies (which contribute to CO_2_ sensitivity) depend on PHOX2b for their development. The postnatal impairment of the hypercapnic ventilatory response seen in *PHOX2b*^*+/−*^ pups resolved before 10 days of postnatal age. Importantly, the chemosensitivity impairment was closely dependent on ambient temperature. Around thermoneutrality, the hypercapnic ventilatory response increased with ambient temperature, although it was still lower in mutant than in wild-type pups. However, the difference between the absolute increase in ventilation in mutant and wild-type pups become more pronounced as the temperature increased above 29°C. At lower ambient temperatures (26°C), the cold-induced depression of the ventilatory response to hypercapnia was less pronounced in mutant pups. Thus, the *PHOX2b* mutation affected not only the control of breathing, but also the interaction of breathing with thermoregulation. In 2-day-old *PHOX2b*^*+/−*^ pups, the immediate hyperpneic response to hypoxia was normal but the hypoxic decline was markedly increased. The ventilatory decrease caused by hyperoxia was larger in newborn *PHOX2b*^*+/−*^ mutant mice than in their wild-type littermates and was magnified by longer apnea durations, which suggested stronger tonic activity of oxygen-sensitive peripheral or central chemoreceptors. Sleep apnoea time was increased about 6-fold in 5-day old *PHOX2b*^*+/−*^ mutant pups and ventilation during active sleep was decreased by about 20%, compared to wild-type pups. Thus, heterozygous Phox2b invalidation produced respiratory phenotypes that differed markedly from CCHS.

#### Constitutive *PHOX2b*^*27Ala/+*^ mice

Mouse models of CCHS were obtained using a knock-in approach to introduce a +7 alanine expansion of the 20-residue polyalanine tract (the PHOX2b^27Ala^ allele) [15]. The heterozygous *PHOX2b*^*27Ala/+*^ offspring of chimeric founders were cyanotic soon after birth and died within a few hours. During the first 20 min after delivery, the breathing pattern ranged from gasping or very unstable breathing interrupted by apnoeas to relatively shallow breathing at a slower rate compared to wild-type littermates. Apneic episodes were more frequent and lasted longer in the mutants compared to the wild-type pups. Importantly, the mutant pups had no response to hypercapnia. Thus, *PHOX2b*^*27Ala/+*^ mice reproduced two characteristic symptoms of human neonates with CCHS: blunted or unstable breathing with apnoeas, and absence of a ventilatory response to hypercapnia. Neuroanatomical studies of *PHOX2b*^*27Ala/+*^ mice revealed that the RTN/pFRG precursors degenerated or failed to differentiate as early as embryonic day 12.5, whereas the pre-Bötzinger complex was functional in embryos.

#### Conditional *PHOX2b*^*27Ala/+*^ mice

A crucial issue regarding CCHS is whether genetic RTN deletion and the associated loss of chemosensitivity in *PHOX2b*^*27Ala/+*^ pups accounts fully for their lethality at birth. The answer is negative, as shown by a second mutant mouse model in which *PHOX2b*^*27Ala*^ is specifically targeted to the RTN [8]. Although the Egr2^cre/+^; *PHOX2b*^*27Ala/+*^ mouse mutants lacked the RTN and their breathing was not stimulated by CO_2_ elevation, they survived to adulthood. Despite the absence of a normal CO_2_ response, PCO_2_ in the blood was within the normal range and perinatal mortality was very low. At 3 weeks of age, the mutants had normal baseline ventilation in room air but increased their ventilation only slightly in response to hypercapnia, to 20% of the control value. At 4 months, adult mutants had significant but incomplete recovery of the ventilatory response to hypercapnia (to 40% of the control value). The mutants showed vigorous and sustained minute-ventilation increases in response to hypoxia, indicating the presence of functional peripheral chemoreceptors. Furthermore, when exposed to 100% O_2_, all mutants had periodic breathing with long apneic pauses, indicating greater dependence on tonic peripheral chemoreceptor function to maintain a normal respiratory pattern.

Neither the RTN nor CO_2_ chemosensitivity is required for life-sustaining breathing, yet constitutive *PHOX2b*^*27Ala/+*^ pups die soon after birth. Thus, defects in other *PHOX2b + *neurons of the reticular formation may contribute to the lethal breathing disorder at birth in these pups.

Taken together, the fact that genetic elimination of the RTN severely abolishes CO_2_ sensitivity in constitutive and conditional *PHOX2b*^*27Ala/*+^ mice supports a role for a similar mechanism in the CO_2_ insensitivity that characterizes CCHS. However, the survival and functional breathing in conditional *PHOX2b*^*27Ala/+*^ mice also suggest that the neurological basis for CCHS is not confined to absence of the RTN and that compensatory mechanisms partially restore CO_2_ sensitivity.

### Overview of CCHS and PHOX2B mutations

***Jeanne AMIEL****. MD, PhD. Necker-Enfants Malades University Hospital, French Centre of reference for central hypoventilation, Paris, France*

CCHS is a rare disorder of the autonomic nervous system with an estimated incidence of about 1 in 200,000 live births [13]. CCHS is characterised by central hypoventilation and the absence of a chemoreflex to CO_2_. It classically manifests in newborn babies. Isolated central hypoventilation can occur later in life, childhood to adulthood. This manifests as nocturnal alveolar hypoventilation with mild autonomic nervous system dysfunction [23]. CCHS can be associated with neurocristopathies, such as Hirschsprung's disease (16% of the cases) [13] and neuroblastoma (5-10% of the cases [24,25]. Neuroblastoma is the most frequent extra-cerebral solid tumour in children [26]. Hirschsprung's disease (HSCR) is a malformation of the enteric nervous system which leads to intestinal occlusion. The incidence of isolated HSCR is of 1 in 5,000 live births and the major disease-causing gene is the RET proto-oncogene [27].

The association of CCHS with neurocristopathies and reports of the condition in monozygotic twins and in the offspring of a woman with CCHS suggested a genetically determined and possibly monogenic condition [28,29].

*PHOX2B* was subsequently identified as the disease causing gene in CCHS and some cases of late onset CHS [4,5,24,30]. Importantly, no *PHOX2B* gene mutations are found in patients with sudden death syndrome or hypoventilation associated with hypothalamic dysfunction.

*PHOX2B* encodes a paired box homeodomain transcription factor, characterized by two polyalanine repeats of 9 and 20 residues in the C-terminal region. Heterozygous in-frame duplications leading to alanine expansion within the normal 20-alanine stretch from +5 to +13 alanines are the most frequent mutations (92%). Other mutations are frameshift mutations leading to aberrant C-terminal region, nonsense mutations and missense mutations within the homeodomain [4,5,12]. In control subjects alanine contractions (−5, −7 and −13 alanines) in the 20-alanine stretch can be observed with no phenotypic consequences reported to date.

In most case the mutation arose de novo in the index case. However, 15 to 20% of unaffected parents show somatic mosaicism for the mutation identified in the child [5]. Mosaic mutations arise from errors during mitotic divisions. When a parent is a mosaic the recurrence risk can be as high as 50% and depends on the degree of mosaicism in germ cells. As a germ line mosaicism cannot be ruled out, parents with no somatic mosaicism detected are counseled at 1% recurrence risk in siblings [31]. An adult with CCHS has a 50% risk of transmitting the mutation to the offspring. Pregnancies of CCHS female reported to date were normal and babies were born around term.

Among alanine expansions, the shortest (i.e. +5 alanine expansion) is incompletely penetrant for the ventilatory phenotype and individuals may develop late-onset central hypoventilation or no symptoms at all. They do not present Hirschsprung's disease or neuroblastoma. Asymptomatic cases harbouring a +5 alanine expansion are at risk of acute ventilatory decompensation during anaesthesia, respiratory infections or some therapies. They, and their doctors, have to be informed of these risks [4,24,30].

From alanine expansions of +6 and above, the ventilatory phenotype is fully penetrant and will lead to CCHS. Individuals with the +6 genotype have a moderate risk and those with +7 or more alanine expansion are at greatest risk for Hirschsprung’s disease. Neuroblastoma rarely occurs in patients with alanine expansions. To date, there is only one report of neuroblastoma which occurred in a patient with a +13 alanine expansion mutation [24].

Around 8% of patients with the CCHS phenotype have heterozygous *PHOX2B* gene mutations that do not lead to alanine expansion but frameshift, nonsense or missense [4,5,12,24,30]. These patients are at high risk of developing neuroblastoma and Hirschsprung’s disease. Frameshift mutations can lead to either a +1 nucleotide or a +2 nucleotides frame. Mutations with +1 nucleotide frameshift, with and without 20-alanine expansion, have a longer protein, while +2 nucleotide mutations have a shorter protein.

The capacity of PHOX2B mutant proteins to transactivate the dopamine beta-hydroxylase promoter, a known target for PHOX2B protein, has been investigated *in vitro*. In the luciferase assay, the potential of PHOX2B proteins with alanine expansions to transactivate the dopamine beta-hydroxylase promoter declines with increasing length of the polyalanine stretch from +6 alanine expansion while the one of the +5 alanine expansion is similar to controls.

Transfection of *PHOX2B* wild-type and mutant constructs in HeLa cells^a^ shows that mutant PHOX2B proteins (≥ + 9 alanine expansion) aggregate in the cytoplasm, which abrogates their mobility and function in the nucleus. In gel filtration studies of *in vitro* translated proteins, wild type proteins form dimers, > + 5 alanine expansion proteins form oligomers while +5 alanine expansion proteins may oscillate between dimeric and oligomeric forms [11] CCHS mutant proteins, therefore, like polyQ proteins tend to misfold. This may explain the loss of transactivation of the dopamine beta-hydroxylase promoter.

The ability of proteins to refold after misfolding was investigated by fluorescence recovery after photo-bleaching (FRAP) of cells transfected with either wild type or mutant fluorescent tagged PHOX2B constructs. The time of recovery was delayed for mutant proteins and was related to the length of the alanine expansion mutations. The longer the alanine expansion the more misfolded and reduced mobility of the PHOX2B mutant protein within the cell. FRAP could be a reliable method for screening drugs that have the ability to refold proteins, and provide a new therapeutic approach to pharmacological treatment of CCHS.

Misfolding can lead to aggregation of proteins. Both processes activate intracellular chaperone proteins such as, heat shock proteins which assist in folding and refolding of proteins and, in preventing protein aggregation. Indeed, chaperone proteins co-localize with aggregates of PHOX2B proteins. Treatment of transfected cells with mutant constructs and the antibiotic geldanamycin, an enhancer of heat shock proteins, increases the mobility of the mutant proteins, and further translocation to the nucleus [32]. Misfolding of PHOX2b proteins may explain the loss of transactivation potential of mutant proteins. Like alanine expansion, shorten or elongated PHOX2B mutant proteins abolish or severely reduce the transactivation of the dopamine beta-hydroxylase promoter and oligomerise even in the absence of a normal 20-alanine tract.

The misfolding of mutant PHOX2B protein is not the only mechanism leading to dysfunction of the ventilatory autonomic system. A premature stop codon mutation in a CCHS patient has led to the production of an N-terminally truncated protein, by re-initiation of translation, which did not form oligomers. The shortened protein was located in the nucleus and e transactivation of the dopamine beta-hydroxylase promoter was similar to controls [33]. The mechanism by which this mutant protein leads to ventilatory dysfunction is unknown.

Complete deletion of the *PHOX2B* gene is a rare event which has been observed in a patient with Hirschsprung's disease and in three families with CCHS [34]. Thus, as heterozygous loss of *PHOX2B* function leads to variable phenotypes, the role of modifier genes is highly likely. This is also true for alanine expansion and frameshift mutations. Note that no *PHOX2B* gene mutations are found in patients with sudden death syndrome [35-37] or hypoventilation associated with hypothalamic dysfunction [38].

*PHOX2B* mutations may trigger a wide range of autonomic nervous system disorders, ranging from congenital malformation to tumour predisposition. Some genotype-phenotype correlations are currently used by practitioners for counselling patients and their families. However, these phenotype-genotype correlations are incomplete and the molecular mechanisms underlying phenotypic variability are still poorly understood. Protein misfolding and oligomerisation occur in the vast majority of *PHOX2B* mutations, either alanine expansion or frameshift mutations. The CCHS phenotype cannot be explained purely by a loss of PHOX2B function.

### Germline mosaicism and de novo PHOX2B mutations in CCHS: implications for penetrance and inheritance

***Tiziana BACHETTI,****PhD. Laboratorio di Genetica Molecolare, Istituto Giannina Gaslini, Genova, Italy*

Genetic testing has confirmed the presence of *PHOX2B* mutations in 93 patients, with congenital central hypoventilation syndrome (CCHS) from within and outside Europe. A wide variety of mutations were identified. While the most frequently reported mutation was a variable expansion of a 20-alanine stretch ranging from +5 to +13 alanines within exon 3 of the *PHOX2B* gene, frameshift and missense mutations also occur but only occasionally within exon 1, 2 or 3 of the *PHOX2B* gene [30,31,39,40]. The presence of a *PHOX2B* mutation confirms the diagnosis of CCHS.

Molecular diagnosis of *PHOX2B* mutations in CCHS patients is performed by PCR amplification and sequencing analysis initially in exon 3 of *PHOX2B* gene [39]. If no mutations are detected, sequencing analysis is undertaken in *PHOX2B* exon 1 and exon 2. Parents of a child with a mutation can request analysis of their own DNA. When a polyalanine expansion is detected, a more specific analysis is performed to identify whether the mutation is a constitutive mutation, which is carried by all cells of the individual, or a mosaic mutation, carried only by a fraction of cells in which the mutation has been acquired after fertilization. In particular, mosaicism is caused when a mutation arises within an individual cell during embryogenesis. Only tissues that derive from that cell will harbour the mutation in the individual. The resulting individual will have a mixture of cells, some with, and some without the mutation. Mosaicism can be germline (affecting egg or sperm cells) or somatic (affecting non-germinal cells). CCHS patients with polyalanine expansions may have a somatic or germline mutation. In a germline mutation, CCHS is transmitted to the offspring if they inherit the PHOX2B allele carrying the mutation as CCHS is autosomal dominant disorder. However, if the individual inherits the wild-type allele, the child will be a healthy individual.

To identify the mosaic transmission of the allele, a sensitive diagnostic method is used which couples amplification of the shorter amplimer spanning only the polyalanine region, with FAM-tagged primers, and capillary electrophoresis. Polyalanine expansion mutations produce two peaks on electrophoresis which represent the expanded polyalanine and the wild-type alleles.

The ratio of the area under the expanded allele (E) and the sum of the area under both wild-type and expanded alleles (E + N) is a measure of polyalanine expansion length. The ratio (E/E + N) decreases with increasing length of the polyalanine expansion. Analysis of *PHOX2B* mutation in parents who are carriers of CCHS show that mosaic carriers have smaller E/E + N ratios (with polyalanine expansions >5 alanine residues), while constitutive carriers have ratios consistent with a +5 alanine expansion.

The constitutive +5 alanine expansion may be completely or partially penetrant, and may lead to CCHS, late-onset CCHS, or an asymptomatic individual. However, constitutive expansions ≥ +6-alanine expansion are completely penetrant. A consequence of this observation is that asymptomatic CCHS parents of a proband carrying the +5-alanine expansion may have a constitutive mutation, while the asymptomatic parents of a proband with expansions longer than +5-alanine expansion have a mosaic mutation. Therefore, +5 alanine expansions are constitutive mutations associated with incomplete penetrance in parents, while >5 alanine expansions are mosaic mutations in asymptomatic parents but may be constitutive mutations in symptomatic carriers.

In our cohort of patients and parents most of the polyalanine expansions are *de novo* mutations with only a small fraction of patients inheriting the illness from a parent with a constitutive or mosaic mutation [31]. Until recently it was thought that about 10% of *PHOX2B* polyalanine expansions were inherited from one parent. However, recurrence risk of CCHS depends on the origin of the *PHOX2B* mutation. In the case of de novo mutations the risk of transmission is very difficult to estimate, because the mutations are very rare. In the case of inherited mutations, if derived from a constitutive carrier, the risk of transmission is 50%, while when derived from a mosaic carrier the risk of transmission is less than 50%.

The detection of low levels of *PHOX2B* mosaicism using both short and long amplimers in the ‘FAM’ and ‘sequencing’ method have been compared [40]. Sequencing was not very efficient in detecting low levels of mosaicism, with both the long, and the short amplimer. The short amplimer detected 30 to 50% and the long amplimer 50% or more mosaicism depending on the length of the polyalanine tract. In contrast, the ‘FAM’ method was much more sensitive: the short amplimer was able to detect all levels of mosaicism for each polyalanine expansions, and the long amplimer was able to detect from 2 to 10% mosaicism which correlated with the respective length of the polyalanine expansion. The short amplimer was more efficient in detecting *PHOX2B* expansion, and the ‘FAM’ method more sensitive than ‘sequencing’ method. The best combination for analysing *PHOX2B* polyalanine expansion levels is therefore to use the shorter amplimer, followed by FAM labelling. Applying this technique to patients’ parents, previously resulted not to carry the proband’s mutation by using the ‘sequencing’ method, increased the transmission of inherited *PHOX2B* mutations from 10% to 25%. In conclusion our data show that, compared to the sequencing method, the ‘FAM’ method is able to detect a lower percentage of mosaicism in CCHS parents carrying polyalanine expansions.

These observations suggest that all CCHS parents, who have been previously analysed by the sequencing method should be re-evaluated using the FAM method to exclude low levels of mosaicism and to better predict the risk of CCHS transmission to the proband. Values of the ratio (E/E + N) obtained by serial dilutions of DNA carrying different length polyalanine expansions provide an estimate of the extent of mosaicism.

The detection of somatic mosaicism in the parent of a child with CCHS is confirmation of the presence of a germline mutation. The risk of parents transmitting a somatic mosaicism is 50% or lower, but it is difficult to estimate because the extent of mosaicism in somatic cells may be different from mosaicism in germline cells. A polyalanine somatic mosaicism in parents may also have implications, if there is incomplete penetrance or late-onset CCHS.

Levels of the mosaicism detected by the FAM method are not definitive because the limit of detection may be improved by new technologies. Some negative parents identified so far by the FAM method could carry a low level of the mutant allele, which is still unidentified.

### Hints on potential future treatments from other homopolymer associated diseases

***Kenneth H. FISCHBECK****. Neurogenetics Branch, National Institute of Neurological Disorders and Stroke, National Institutes of Health, Bethesda, Missouri, USA*

Polyglutamine (polyQ) disorders, such as Huntington’s and Kennedy’s disease [41], and polyalanine disorders, including CCHS [42], are all single amino acid repeat expansion conditions. They may therefore share some important underlying pathological mechanisms that lead to neuronal loss. The lessons learned from research into polyQ disorders, including the challenges of developing effective treatments in humans, may well be applicable to CCHS.

Huntington’s disease (HD) is one of at least nine different polyQ neurodegenerative disorders. It results from an autosomal dominant mutation which causes abnormal involuntary writhing movements called chorea. It usually presents in patients over the age of 30 years. The symptoms include psychological changes, depression, impulsive behaviour and cognitive decline. There is widespread loss of neurons in the central nervous system, particularly in the cerebral cortex and basal ganglia. Most vulnerable are the striatal medium spiny neurons in the caudate nucleus. The mutation is a CAG trinucleotide repeat expansion of 40–60 or more CAGs compared with an average of 20 CAGs in normal individuals. The product of the mutant HD gene is called huntingtin, and its function is unknown [41,43]. Cell culture and animal studies indicate that it may play a role in vesicular transport or in the regulation of transcription [44].

Kennedy’s disease is an adult-onset, X-linked motor neuropathy, also known as spinal and bulbar muscular atrophy. It leads to weakness of the face, tongue and the pharyngeal muscles, as well as the extremities. Respiratory muscles may also be affected and, like CCHS patients, those with Kennedy’s disease may develop CO_2_ retention and respiratory failure. Only men are fully affected by this disease; female relatives are generally asymptomatic [45,46]. In contrast to Huntington’s disease, the normal function of the gene product, the androgen receptor protein, is well known.

Kennedy’s disease is caused by an expansion of a CAG repeat in the first exon of the androgen receptor gene. CAG repeats in the mutant AR gene range from 40 to 62 CAGs, whereas normal individuals have 10 to 36 CAGs [41,43]. There is a correlation of repeat length with disease severity; the longer the repeat, the earlier the onset and the more severe the disease [47].

The androgen receptor (AR) is a nuclear hormone receptor, and in the absence of ligand it is found in the cytoplasm. It has a transactivation domain with a polyglutamine tract, and DNA and ligand binding domains. When a hormone ligand (testosterone or dihydrotestosterone) binds to the AR, the activated receptor undergoes a conformational change, dissociates from heat shock proteins, and partners with another AR receptor to form a dimer. The AR dimer binds to a specific sequence of DNA and interacts with other proteins in the nucleus to up- or down-regulate specific targets [48].

Ligand binding is unaltered by the repeat expansion [49]. As with *PHOX2B* in CCHS, there is abnormal target gene activation by the mutant AR protein. There is some loss of AR function in patients with Kennedy disease, as manifested by signs of androgen insensitivity [50]. However, studies show that loss of AR function alone, as in androgen insensitivity syndrome [51] does not cause the motor neuron degeneration that occurs in Kennedy disease.

The primary effect of the repeat expansion is a toxic gain-of-function in the mutant AR protein. The protein misfolds and has the tendency to form aggregates. These aggregates (or at least the tendency of the mutant protein to aggregate) may be toxic to the motor neurons in the brainstem and in the spinal cord, causing progressive motor neuron degeneration. This toxic gain-of-function has been demonstrated in experimental models and is the likely mechanism for neurodegeneration which leads to selective neuronal loss in this and other polyQ disorders [52].

The toxicity of the AR protein is related to the length of the expanded polyQ tract and is greater in the nucleus than in the cytoplasm [53]. The mutant AR protein binds to other proteins in the nucleus, depleting critical nuclear factors, which may lead to transcriptional dysregulation with altered histone acetylation [54].

Neurons are protected from the effects of the AR mutant protein by the ubiquitin–proteasome pathway and by heat shock proteins, which serve as molecular chaperones. Misfolded proteins and their aggregates are targeted for degradation by the ubiquitin–proteasome pathway, while chaperone proteins assist in refolding, dissembling, and reassembling proteins. Neuronal inclusions of mutant protein in mouse models and human tissue stain positively for components of the ubiquitin–proteasome pathway [55-58]. This pathway degrades truncated fragments of the androgen receptor, and molecular chaperones such as HSP90 may target the mutant proteins for degradation [59]. Inclusions of mutant proteins are observed not only in the nucleus but also in the cytoplasm of cultured neuronal cells [60] in transgenic animal models [61] and in autopsy material [62]. It is now thought that inclusions of mutant proteins may reflect a protective response by the cell to the toxic protein.

As the mutant AR protein is toxic in the presence of androgen, drug treatments that reduce androgen levels, such as leuporelin and dutasteride, have been investigated in humans following promising results in animal models. In humans, leuprorelin only had a significant effect on swallowing in a post hoc analysis of patients who had been symptomatic for less than 10 years [63]. While quality of life improvements were observed with dutasteride, there were no significant effects on muscle strength [64].

There are on-going research programs investigating AR modulation in animal models, the effect of geldanamycin derivatives on the protective response of heat shock proteins, the efficacy of curcumin derivative ARCJ9, and the effectiveness of insulin-like growth factor-1 (ICG-1) on neuroprotection and muscle regeneration.

PolyQ disorders, unlike CCHS, are late-onset, progress very slowly, and a large patient sample or a long follow-up period is required to test the efficacy of treatments on disease progression. Both mouse models and clinical studies suggest that for treatments to be effective, they must be started early in the course of the disease. To achieve this, widely available genetic tests, relevant therapy targets, and reliable clinical outcome measures are required, as well as biomakers to assess the likely effectiveness of treatment.

Potential new therapeutic targets for investigation include agents that block gene expression and intercellular transport of mutant proteins, especially those that enhance the protective mechanisms that degrade the mutant protein and those that mitigate the toxic effects of mutant protein by correcting transcription and signal transduction defects.

Cell culture models offer assay systems for screening chemical compound libraries, with medicinal chemistry to optimize the hits. Biological approaches including oligonucleotides that reduce RNA transcripts of the mutant gene are under development for these and other neurodegenerative diseases.

In conclusion, to develop effective treatments for these diseases researchers need to address the following: (i) Is it a loss-of-function or a toxic gain-of-function mechanism; (ii) Is it a gene that needs to be replaced or one that needs to be knocked down; (iii) Is it a developmental disorder that can only be treated before birth or in early infancy, or is it a condition with an on-going toxic effect of a mutant protein, or the effect of a deficiency in the mutant protein that might be amenable to treatment later in life, after diagnosis.

Important needs include a network of clinicians and patients for clinical studies, clinical protocols with relevant outcome measures and family groups available for study, and early involvement of pharmaceutical companies to make use of their expertise and knowledge in developing effective treatment.

### Desogestrel: facts and hope

***Ha TRANG, Thomas SIMILOWSKI, Christian STRAUS****. French Centre of Reference for Central Hypoventilation, Robert Debre University Hospital and La Pitié Salpêtrière University Hospital, Paris, France*

The observations that the response to CO_2_ was restored in two female patients with CCHS taking desogestrel as a contraceptive pill has led to hypothesis that progesterone may be effective in stimulating the breathing response. As a result, a clinical study to evaluate the effect of the drug on the respiratory response to CO_2_ in CCHS patients is currently in progress^b^. We review here the cases of the two female patients with neonatal CCHS, who at puberty received desogestrel as a contraceptive pill and hypothesize on the mode of action of desogestrel in restoring ventilatory response to CO2.

Both female patients were diagnosed with CCHS at birth and followed-up since then in the French Centre of Reference of Central Hypoventilation. The first patient was 19 years old at the time she was taking desogestrel. She had a +5 alanine expansion on the *PHOX2b* gene. Since birth she had hypoventilated and required mechanical ventilation during the night. In 2006, she had no respiratory response to the exogenous CO_2_ challenge test. Three years later, when the test was repeated, there was a dramatic increase in respiratory rate and volume. On taking her medical history, it was revealed that she been taking desogestrel, as a contraceptive treatment for one year. The second patient was 30 years old at the time it was observed that her ventilatory control had improved. Like the previous patient she required mechanical ventilation during the night. She had a +6 alanine mutation in the *PHOX2B* gene. In July 2009, she had not responded to the CO_2_ challenge test. At this time she asked for a contraceptive and, in December 2009, received desogestrel. Three weeks later, a CO_2_ challenge test was performed which revealed a marked increase in respiratory and ventilation rates in response to increase of inspired CO_2_. Both patients showed a marked recovery in response to CO_2_; the only feature they shared was treatment with desogestrel.

Following these observations, a literature research revealed only one report in which a pregnant CCHS patient was studied. During pregnancy which is physiologically associated with an explosion in progesterone and estrogens secretion, there was no change in her chemosensitivity to CO_2_, oxygen or response to hypoxia. In the author’s cohort, those CCHS patients using contraceptives, other than desogestrel, do not show evidence that these drugs affect chemosensitivity to CO2.

From these observations, it is hypothesised that desogestrel is a potent progesterone receptor agonist that induces changes in respiratory control by activating autonomic CO_2_ chemosensitive regions that are unaffected by *PHOX2B* mutations, such as chemoreceptors in the hypothalamus and the peripheral nervous system. There is evidence that progesterone can enhance ventilatory response to hypercapnia, and animal studies which indicate that progesterone acts in the brain.

### Keynotes on basic sessions

***Isabella CECCHERINI,****PhD. Laboratorio di Genetica Molecolare, Istituto Giannina Gaslini, Genova, Italy*

This summary is dedicated to those with CCHS and to their families. It outlines our basic understanding of the condition which has allowed us to raise patient’s expectation of care and helped to impact the quality of their lives. The advancement in our knowledge of CCHS is reviewed, in simple terms, starting with the underlying genetic and pathological origins of the illness, and finally addresses the developments in pharmacological treatments for CCHS.

For many years, CCHS was considered a lethal genetic disorder, until medical advancements allowed patients to have their own families. CCHS is a disease which recurs in families, with a 50% risk of transmission from affected parents to their offspring. The birth of a CCHS patient from families without any history of the disease cannot be avoided or prevented. Mutational events occur randomly and rarely, at each gene locus and at each cell division. The chance that the *PHOX2B* gene is affected by a new mutation is very low, but not negligible. Therefore, the birth of a child with CCHS in families without any history of the disease will occur. If new mutational events cannot be prevented and their occurrence cannot be avoided, recent methodological approaches can help predict the risk of recurrence, once *PHOX2B* mutations and somatic mosaicism are identified in patients. *De novo* mutational events cannot be avoided. In such families, when a child is born with CCHS, genetic counseling can help both parents and their children, cope with the condition and understand the risk of transmitting CCHS to any future children.

*In vivo* and *in vitro* studies have deepened our understanding of the pathogenic mechanisms involved in CCHS. They have identified the neuroanatomic lesions underlying the impaired perinatal hypercapnic response, and characterised the aggregates of misfolded PHOX2B proteins and their effect on cellular response. PHOX2B proteins also interact with other proteins regulating gene expression and cell signaling, thus accounting, once defective, for not only the respiratory phenotype, but also the development of accompanying disorders such as neuroblastoma and Hirschsprung disease. These are a few of the advances that have been made.

And finally, the increase in ventilatory responses to hypercapnia observed in two patients using desogestrel raises hope for a pharmacological approach in CCHS. Further clinical research studies should be undertaken to that purpose. Pharmacological therapies developed for polyglutamine disorders may be effective in CCHS. *In vitro* screening of specific drugs has already demonstrated a reversal of mutation-impaired PHOX2B function. I would like to thank all the speakers who have contributed to this interesting session today, and also to all the families who passionately followed our discussions.

### Hypoventilation syndromes: it’s not just about the timing

***Debra E. WEESE-MAYER****, M.D. Autonomic Medicine in Pediatrics (CAMP), Ann & Robert H. Lurie Children’s Hospital of Chicago. Northwestern University Feinberg School of Medicine, Chicago, Illinois*

Respiratory and Autonomic Disorders of Infancy, Childhood and Adulthood (RADICA) are a group of rare diseases that have varying presentation, combined with a range of severity of respiratory control and autonomic nervous system dysfunction. Among these conditions, CCHS and Rapid-onset Obesity with Hypothalamic dysfunction, Hypoventilation, and Autonomic Dysregulation (ROHHAD), have the most severe respiratory phenotypes and require life-long support with artificial ventilation as a mainstay of care. The cause of ROHHAD is not known at the present time. But studying a condition with a related phenotype, such as CCHS, may enable a better understanding of ROHHAD and the control of breathing within the autonomic nervous system dysregulation. This presentation briefly reviews the phenotypes of these two disorders.

In 1970, the first case of CCHS was reported by Robert Mellins and colleagues. More than 30 years later, in 2003, *PHOX2B* was identified as the disease-defining gene for CCHS. Approximately, 90% of CCHS patients are heterozygous for polyalanine repeat expansion mutations (PARMS) in the second polyalanine repeat region of the *PHOX2B* gene. These expansions have between 24 and 33 alanines in exon 3 of the *PHOX2B* gene on the affected allele (and 20 alanines on the unaffected allele). So CCHS-related *PHOX2B* genotypes are 20/24 to 20/33. The remaining 10% of CCHS patients have a non-polyalanine repeat expansion mutation (NPARM) in exon 2 or exon3. NPARMs are usually missense, nonsense or frameshift mutations.

Since 2010, with publication of the 2^nd^ American Thoracic Society (ATS) Statement on CCHS, a *PHOX2B* mutation is required to confirm a diagnosis of CCHS. Over 1,000 *PHOX2B* mutation-confirmed cases of CCHS have been reported worldwide and the most common genotypes are 20/25, 20/26 and 20/27 PARMs. In addition to abnormal respiratory control, CCHS is also associated with a predisposition to Hirschsprung disease, neural crest tumours, cardiac rhythm dysfunction, characteristic facial features, ophthalmologic abnormalities, and more in terms of autonomic dysregulation.

The severity of hypoventilation, autonomic symptoms, and predisposition to neural crest tumours increases with the length of the polyalanine repeat expansion. Individuals with 20/26 to 20/33 PARMs and those with NPARMs have a higher risk of Hirschsprung disease. Patients with PARMs 20/29-20/33 and, especially those with an NPARM, have a predisposition to tumours of neural crest origin. Neuroblastoma occurs primarily in NPARMs, while ganglioneuroma, ganglioneuroblastoma and occasionally neuroblastoma develop in a small subset of those with the longest PARMs.

Children with CCHS have cardiovascular, respiratory and ophthalmologic symptoms consistent with autonomic nervous system dysfunction. Holter monitoring in CCHS subjects shows a positive correlation between the number of alanine repeats and longest r-r interval duration (≥3 secs). Patients with a 20/27 genotype are at greatest risk of cardiac pauses (83%), and more likely to receive a cardiac pacemaker than those with the 20/25 or 20/26 genotypes (0% and 19%, respectively). Failure to receive a pacemaker when the r-r interval is ≥3 secs may lead to death or severe neurocognitive defects.

Respiratory control is impaired in CCHS, and individuals with NPARMs typically have a more severe continuous ventilator dependence than those with PARMS. During sleep, ventilatory sensitivity to endogenous hypercapnia, hypoxia or the combined stimulus is negligible or absent. So individuals with CCHS will not increase their breathing in response to these physiologic stimuli, nor will they spontaneously arouse from sleep. With awake exogenous challenges of combined hypercapnia-hypoxia, virtually all patients with CCHS, irrespective of genotype, have a delayed response with a modest but insufficient increase in minute ventilation. This residual responsiveness to a hypercapnia-hypoxia challenge suggests that pharmacologic interventions may be effective in recovering chemosensory function and the perception of asphyxia.

Pupillometry studies with a handheld pupillometer have demonstrated impairment in both sympathetic and parasympathetic nerve function in CCHS patients when compared with normal control subjects. There is an inverse linear relationship in the diameter, constriction and dilation velocities of a patient’s pupillary response to light and length of the most common *PHOX2B* PARM genotypes. This suggests a graded CCHS phenotype/*PHOX2B* genotype response based on polyalanine repeat length, which may provide an objective outcome measure in intervention trials.

Patients with CCHS have characteristic facial features that differ from matched control subjects. Facial photogrammetry of midface and lip (lip trait is the overturned lateral one-third of the upper vermilion border, so flesh-colored instead of pink) trait variables correctly predicted 85.7% of CCHS cases and 82.2% of control subjects. Severity of these facial features, including flattening of the face and shortening of the upper and midface, increase with the length of the *PHOX2B* alanine repeat expansion.

At present there is no correlation between neurocognitive ability of school-age children with CCHS and specific *PHOX2B* genotypes.

Later-onset CCHS (LO-CCHS; individuals ≥ one month of age) typically have the *PHOX2B* 20/24 and 20/25 genotypes, and may not be identified until well into adulthood. They present with a variety of symptoms, including abnormal responses to carbon dioxide and oxygen challenges. Hirschsprung disease and tumours of neural crest origin are rare. Cardiac asystoles can occur in adults with LO-CCHS, but thus far have not been reported in children. Neurocognitive function may be impaired in those not identified until adulthood, but specific testing of children with LO-CCHS has not been reported. While children with LO-CCHS have the characteristic CCHS-related facial features, the facies are more difficult to identify in adults especially if they have a moustache that disguises the “lip trait”.

Patients with *de novo* deletions of the *PHOX2B* gene present with variable phenotypes, including total gut aganglionosis, ganglioneuroblastoma, swallowing difficulties and apparent life-threatening events. These children generally lack significant portions of the *PHOX2B* gene and several other nearby genes. The condition has an autosomal dominant inheritance pattern but may occur *de novo*. The expectation with the heterozygous condition and absent *PHOX2B* gene, would have been one of haploinsufficiency.

In contrast to CCHS, the onset of ROHHAD occurs after 1.5 years of age in otherwise seemingly healthy children. Early recognition and treatment of the symptoms associated with ROHHAD are essential as there is a high prevalence of cardio-respiratory arrest. At present, there is no diagnostic test, and no defining gene has been identified. The disease is not widely recognized by healthcare professionals, though with recent efforts nearly 100 cases have been reported. Forty percent of these cases present with ganglioneuroma or ganglioneuroblastoma.

The ROHHAD phenotype evolves with advancing age and is characterized by rapid-onset obesity in the first 7–10 years of life, followed by hypothalamic dysfunction, onset of symptoms of autonomic dysregulation and later onset of alveolar hypoventilation. Obesity is accompanied by growth deceleration, and weight loss does not lead to reduced hypoventilation. Children with ROHHAD typically have an absent or attenuated response to hypoxia and hypercapnia, though not formally reported. There is a wide variation in the interval between the onset of hypothalamic dysfunction, autonomic dysregulation and hypoventilation, and the severity of the phenotype evolves with advancing age.

Ophthalmologic defects manifest as increased dilation (mydriasis) and decreased contraction (myosis) of the pupil to light stimuli when compared with control subjects, suggesting sympathetic and parasympathetic neuronal dysfunction. Thermal regulation is also impaired in ROHHAD patients which manifests as peripheral extremity hyperthermia at night and peripheral extremity hypothermia during the day, indicating altered vasomotor tone and temperature dysregulation.

ROHHAD patients may have changes in behaviour and mood, and the subset that has experienced a cardio-respiratory arrest may have significant neuro-developmental impairment. In a subset of individuals with ROHHAD, the Intelligence Quotient (IQ) score is reduced, likely reflecting those who experienced an arrest or those whose hypoventilation went unnoticed and without adequate ventilatory support. However, a remarkably high percentage of children with ROHHAD have normal or high IQs (above 120). At present, treatment for ROHHAD is symptom-based as for CCHS. The primary goal is to optimize the neurocognitive potential of these children. Care requires the services of a multidisciplinary team, parental support and greater awareness of the condition among all healthcare professionals—especially the general paediatrician who will identify the rapid weight gain on the plotted growth curve at a well-child visit.

Both CCHS and ROHHAD are diseases of the autonomic system and have severe respiratory phenotypes. However, *PHOX2B* mutations are not present in patients with ROHHAD. This finding indicates that a different genetic pathway is involved in the differentiation or development of the ROHHAD phenotype.

Sharing information on CCHS, ROHHAD and other disease of RADICA may help us to understand the control of breathing within the autonomic nervous system and the genetic pathways that lead to the development of these conditions, in addition to understanding the basic underpinnings of respiratory control and autonomic regulation in healthy children.

### What’s new in ventilatory support?

***Matthias FRERICK****, MD. Klinikum Dritter Orden, Munich, Germany*

Hypoventilation is the hallmark of CCHS and therefore life-long artificial ventilation delivered either by positive or negative pressure ventilation is needed [65]. Positive pressure ventilation may be delivered by invasive or non-invasive means; while negative pressure ventilation is mostly provided by breathing pacemakers. This article briefly reviews the outcomes of long term home mechanical ventilation (HMV), the performance of ventilatory devices and the socio-medical aspects of long-term ventilation.

Studies about HMV have investigated the clinical setting for the initiation of HMV and the reasons for re-admission to hospital after extended use of the ventilators. A study comparing 28 patients with nocturnal hypoventilation randomised to receive either initiation of HMV as outpatients, or as inpatients found that there was no difference between the two groups for blood gas tensions, ventilator compliance, healthcare professional contact time, and time in hospital. Outpatient initiation of HMV was, therefore, as effective as standard inpatient procedures [66].

After initiation of HMV via tracheostomy in 109 paediatric patients with chronic respiratory failure, 44/109 (40%) patients were re-admitted to hospital within 12 months. Fifty percent of admissions occurred during the first three months after index discharge. The reasons for re-admission included cannula related problems, infections of the airways and lung. Only a change in the child's treatment before discharge was associated with re-admissions shortly after index discharge, indicating that stability of the condition before discharge is highly important. In order to manage HMV users’ expectations, it is important that all those who use or administer HMV support, carers, patients, and parents, are aware of the possibility of re-admission to hospital [67]. An assessment of the remoteness of the health centre (100 km) from HMV-dependent children and their family as well as their level of education do not appear to affect mortality rate and patient outcomes [68].

Prolonged positive pressure ventilation carries potential complications, including diaphragm atrophy. This has been observed in both animal and human studies. Levine et al. [69] compared diaphragm biopsies of 14 brain dead organ donors with intraoperative diaphragm specimens from 8 control subjects undergoing operations for chest problems. Diaphragmatic inactivity and mechanical ventilation was sustained for 18 to 69 hours in the case subjects and for 2 to 3 hours in control subjects. After only 18 hours of positive pressure ventilation the diaphragm of case subjects showed marked atrophy. Compared to control biopsy diaphragms, the specimens from case subjects showed a 57% decrease in the area of slow twitch fibres and a 53% decrease in fast twitch fibres. Diaphragm muscle atrophied faster than skeletal muscle. Inactivity of the diaphragm led to oxidative stress and increased proteolysis resulting to muscle atrophy, and was not due to inflammation.

Bench tests are used to assess the performance of non-invasive home mechanical positive-pressure ventilators (NI-HMPPV) using settings representative of common respiratory insufficiencies. In a study of 17 non-invasive positive-pressure ventilators in which assisted control, pressure support ventilation or both, if available, were tested, no single ventilator was able to adequately ventilate six common paediatric ventilatory patterns. Only few ventilators were able to ventilate the youngest patients. The sensitivity of the flow and pressure triggers were insufficient, although the pressure trigger was better with a closed circuit, and the flow trigger worked better with open systems [70]. Physicians should be aware of ventilator performance when selecting a HMV for patients.

A lung bench test study assessing the maintenance of minimal tidal volume by NI-HMPPV has compared the performance of 6 ventilators during baseline alveolar hypoventilation, and hypoventilation associated with an increase in airway resistance, a decrease in lung compliance, and non-intentional leaks. The six ventilators maintained a minimal tidal volume during an increase in airway resistance and a decrease in lung compliance. The maintenance of a minimal tidal volume during a non-intentional leak was associated with large variations in tidal volume and patient-ventilator dyssynchrony. Some devices showed an overshoot of the tidal volume after the leak was closed, which may lead to fragmentation of the sleep, or poor ventilator tolerance. This outcome is also reported in other studies including ventilators using double circuits [71].

Volume-assured non-invasive positive pressure ventilation is a novel mode in which circuit leaks have little effect on the delivered respiratory minute ventilation. However, these ventilators are not currently available for HMV [72].

In a study of the user friendliness of HMVs, physicians in ICU, without practical experience of HMVs, were assessed on their ability to operate 11 HMVs. Compared to trained technicians, physicians were slower to operate the ventilators and made mistakes, suggesting the need to standardise the ergonomics of these machines, and to train ICU physicians to use them [73].

Computer control of mechanical ventilators using artificial neural networks (ANNs) may increase the safety of mechanical ventilation. In principle ANNs offers significant advantages over the rule-based system used by HCPs, as ANNs have the potential to learn and adapt to the individual patient's response. An initial study has compared settings chosen for 10 virtual patients by 10 clinical experts. Not only did the expert’s choice of setting all concur; 10 of the 40 ANNs were also able to control the ventilator. In the future, ANNs may have a role in optimising ventilator settings and helping physicians resolve ventilator anomalies [74].

In contrast to positive-pressure ventilation, negative pressure ventilation delivers air in a physiological way to the lungs and has been shown in surfactant depleted rabbits to provide better oxygenation and cause less lung injury [75].

Negative pressure ventilation by phrenic nerve pacing allows patients freedom from the mechanical ventilator during the day [76,77]. The common complications of phrenic pacing include re-operation due to damage of implanted components [78], although these are decreasing due to improvements in device components; receiver pocket infection; axonal damage of the phrenic nerve has been reported in one patient after 13 years of pacing. Phrenic pacing in patients with spinal injuries leads to improvements in quality of life [79-81]. Compared to mechanical ventilation, phrenic nerve stimulation leads to significant reduction in respiratory tract infections [80]. New devices for direct diaphragm pacing are currently under evaluation.

In 2005, the EuroVent Survey found wide variations in HMV provision throughout Europe [82]. To investigate this, Dybwik et al. [83] undertook focus group studies, on HMV treatment, with a wide range of HCPs, which included nurses, pulmonologists and paediatricians across Europe [83]. The most important factor in determining HMV provision was the enthusiasm of skilled HCPs. Attitudes of HCPs, rather than resources, appear to be the pivotal driving force for access to HMV.

Medical problems are also social problems and affect children, parents and caregivers in different ways. Parents and caregivers find living with HMV-dependent children stressful and feel that their child’s life is not valued by society. Experiencing this pressure and problems parents felt it made them a better and stronger person. In contrast, HMV children view the ventilator and hospitals as normal part of their lives, friendships were more important than their medical problems [84]. Nevertheless children hide aspects of their illness from the outside world to avoid isolation and rejection by their friends and society.

Sociological frameworks such as Goffman's theory of ’Stigma‘ may help HCPs to understand the social problems encountered by HMV-dependent children [85]. These children are often discriminated against, unvalued, discredited and isolated. HCPs should support and foster a socialization processes that eliminates stigmatizing language and stereotypes.

In conclusion, all HMV modalities are still ‘state of the art’ and provide the best available support for CCHS patients. Prior to discharge and HMV, patients need to be medically stable. Bench tests show the strengths and weaknesses of ventilators and users of ventilators must know their limitations. New technology may help to optimise ventilator settings. Direct diaphragm pacing is new, and may be an option in CCHS. HCPs need to address both medical and social problems of their patients to enable them to have a better quality of life.

### Criteria for cardiac pacing in children and the general population

***Katarzyna BIEGANOWSKA****, MD. Department of Cardiology, Children’s Memorial Health Institute, Warsaw, Poland*

In 1958, the first cardiac pacemaker was implanted into a middle aged man. Nearly four years later the first pacemaker was implanted into a seven year old boy post operation of tetralogy of Fallot because of syncopes associated with bradycardia. Bradycardia (bradyarrythmia) may cause chronic or intermittent low cardiac output, and patients may present pre-syncopal or syncopal attacks, exercise intolerance and progressive heart failure. In general, the incidence of pathological bradycardia rises with age of the patient.

Bradyarrythmia is the main indication for permanent cardiac pacing and may present as atrioventricular heart block or sinus node dysfunction.

Atrioventricular block may be congenital or acquired mainly post congenital heart diseases operations. In this condition, the sinus node works normally but ventricular rate is dependent on the escape rhythm, which is much slower, atrio-ventricular dissociation is present (no correlation between the sinus and escape rhythms).

Sinus node dysfunction (also known as sick sinus syndrome - SSS) describes a range of heart rhythm disorders, which include bradycardia (sinus bradycardia, sinoatrial block, sinus arrest), tachycardia and bradycardia-tachycardia. SSS occurs rarely in infants and children and is usually due to congenital heart abnormalities. Risk factors for SSS include symptomatic bradycardia, frequent sinus pauses, pre-syncope, syncope, heart failure, symptomatic chronotropic incompetence, inadequate heart rate response to physical activity and symptomatic sinus bradycardia resulting from drug therapy.

In some patients, incidences of bradycardia and symptoms are transient and may be detected only by long-term ECG monitoring. Commonly used noninvasive methods such as ambulatory 24-48-hour Holter ECG recording, transtelephonic ECG or event Holter allow for correlating symptoms with heart rhythm only if the symptom of interest occurs during monitoring. The PocketECG a new technological solution allowing for continuous, noninvasive, real-time ECG monitoring, became available recently. The implantable Loop Recorder ECG (ILR) is an invasive method for registering heart rhythms and is particularly useful when symptoms are infrequent or when aggregate long-term data is required.

In general, the indications for pacemaker implantation are similar in children, adolescents and adults. In 2008, the American College of Cardiology, the American Heart Association, and the Heart Rhythm Society jointly published guidelines for device-based therapy of cardiac rhythm abnormalities [86]. They divided the indications for pacemaker implantation into three specific categories, Class I, (disturbances requiring permanent cardiac pacing, provided the dysfunction was not transient), Class II (permanent pacing is reasonable to performed) and Class III (permanent pacing should not be performed). This year the European Society of Cardiology’s (ESC) new guidelines on cardiac pacing and cardiac resynchronization therapy were published, the main indications are similar to those of previous guidelines.

### Class I

Permanent cardiac pacing is indicated in children for advanced second- or third-degree atrioventricular block associated with symptomatic bradycardia – pre-syncope, syncope, ventricular dysfunction or low cardiac output, in children for advanced second- or third-degree atrioventricular block lasting longer than 7 days after cardiac operation.

A pacemaker implantation should be performed in children with congenital atrioventricular block and with one of the risk conditions: a wide QRS escape rhythm, or complex ventricular ectopy, or prolonged QTc interval, or ventricular dysfunction or with very slow ventricular rate (<50/beats per minute or pauses >3 times the basic cycle length). Permanent cardiac pacing is indicated in children with SSS and correlation of symptoms during bradycardia. Cardiac pacemaker implantation is also indicated for advanced second-degree or complete AV block associated with neuromuscular diseases, for second-degree AV block with symptoms, for asymptomatic patients with average ventricular rate of ≤40 bpm or faster if cardiomegaly or LV dysfunction is present or if the site of block is below the AV node (wide QRS complexes). The procedure is also necessary in adults with SSS and symptoms attributed to bradycardia (for example atrial fibrillation and bradycardia with one or more pauses of ≥5 seconds) and in those with acquired atrioventricular block (irrespective of symptoms for example after catheter ablation of the atrioventricular junction).

### Class II

Permanent cardiac pacing is reasonable in children with congenital heart disease and sinus bradycardia for the prevention of recurrent episodes of bradycardia mediated tachyarrhythmias or with a resting heart rate <40 bpm and cardiac pauses >3 seconds. In children post cardiac surgery with bifascicular block complicated transient compete heart block.

In patients with long QT syndrome, bradycardia and symptoms in spite of B-blockade, left stellate ganglionectomy or implantable cardioverter-defibrillator should be considered rather than pacemaker implantation.

The children with neurocardiogenic pre syncope and syncope as a result of transient autonomic nervous system dysfunction, which is likely resolve by the age of 18 years usually don’t need permanent cardiac pacing therapy.

Patients with CCHS who present with life-threatening brady-arrhythmias and asystoles longer than 3 seconds from associated autonomic dysfunction and chronic hypoxia will require a pacemaker implantation. For patients without cardiac abnormalities, 24-72-hour Holter ECG monitoring is recommended once a year to estimate heart rhythm and exclude brady-arrhythmias, long sinus pauses and asystoles.

The mode of permanent cardiac pacing depends on individual patient characteristics. The system may have either a single or dual chamber leads with endocardial or epicardial pacing. Single chamber devices have one implanted pacing lead in right atrium or ventricle, and dual chamber, two implanted pacing leads (one in the right ventricle and one in the right atrium). These leads subsequently are connected to a pacemaker placed subcutaneously or sub muscles in the chest.

Epicardial pacing is preferred in neonates and children with a weight of <15 -20 kg. It is also used in some children after cardiac surgery and in those without appropriate venous accesses to the heart.

The longevity of cardiac pacing is limited to around 6–8 years. Epicardial lead implantation by thoracotomy can lead to postpericardiotomy syndrome, some patients need pericardiocentesis to remove pericardial fluid and to prevent cardiac tamponade which may be a life threatening complication. Complications occur in both epicardial and endocardial pacing. Lead related complications include fracture, failure of the wire insulation, dislocation, malposition and diaphragmatic stimulation. Lead-related problems such as increased pacing thresholds and decreased impedance can cause premature battery depletion. Cardiac arrhythmias may occur after pacemaker implantation. Pacemaker infections and progressive heart failure can be life-threatening problems. Local pocket-related complications, such as haematoma and venous thrombosis may also be observed post pacemaker implantation. Failed leads need to be replaced. Transvenous extraction of non-functional, abandoned endocardial leads is a difficult and dangerous procedure. Current practice is to remove damaged leads.

Permanent cardiac pacing helps to normalise heart rhythm and improve quality of life, but long-term pacing is limited by the battery depletion and by complications (mainly lead failure).

### Life as an adult

***Linda MIDDLETON****. Parent and UK CCHS Support Network*

Ryan, my son, a young adult, living in UK, has CCHS. He has agreed to me sharing his experience of life as an adult. Some of the issues these young adults face are common to those of any young person, while others are frustrations which are intensified by their medical condition.

After finishing school, Ryan attended a mainstream college and lived at home. He did not want to attend a college for children with special medical needs. Had he wanted to move away from home, we would have supported him, although this would have been difficult. Colleges and universities expect students to be independent in all areas of their lives, and provide much less support than schools. Student accommodation is not usually appropriate for people with medical equipment and who need overnight care.

The medical world is not ready for adults with CCHS in UK. Many local hospitals, including our own, are not familiar with CCHS and ventilators. This makes the transition of medical care, from paediatric to adult services, difficult. In contrast to paediatric services, many adult hospital services may not understand the need for ventilators at night and for full physiological sleep studies. Under children’s services we were used to full physiological sleep studies including recording of oxygen saturation, pulse waveforms, breathing movements, transcutaneous and end-tidal CO_2_, ECG and heart rate, airway pressures and video. Although Ryan always wears a medic alert bracelet, we would want a family member present, if he was admitted to hospital in an emergency. Families need to search for hospital centres that understand the condition and can provide appropriate support.

Public transport, driving a car and traveling present obstacles for these adults; some are refused a driving license. Buses and trains do not provide consistent facilities, security procedures at airports and transporting medical equipment make journeys, very challenging. Although Ryan is now 22 years old, we still take him to and from work (due to unreliable local transport) and collect him from social outings because we feel he is extremely vulnerable.

In his late teens Ryan preferred to go on holiday with his older brother, rather than with his parents. He will decline to go on holiday with friends. Ryan has never stayed away from home overnight without either a member of his family or a carer. He does not want his friends to know he needs a carer to look after him at night. He does not believe his friends would think any less of him, if they knew. However, taking an extra person on holiday to support him at night would be too traumatic.

Ryan is reluctant to tell friends and work colleagues about his condition for fear of being seen as different. He only tells people what they need to know. From a parent’s perspective, the more people who know what to look out for if he becomes unwell and the safer Ryan is. Ryan’s standpoint is different: he is a well person until he goes to sleep and needs a ventilator. As he is with his friends when he is awake, they don’t need to know anymore. The embarrassment and trauma of telling others of their condition is illustrated by a young male CCHS adult who is considering finishing his relationship with his girlfriend, because he does not want to tell her about his medical condition.

Sport is Ryan’s biggest hobby. Football and tennis are his favourites. He was chosen to play in a football match. Once the club knew about his condition, he was excluded. Ryan regrets sharing the information and sees the club’s decision as discrimination. He has left the club and will not join another one.

Ryan understands the risks of taking drugs and alcohol and abstains from them completely. However, there is always the risk of either direct or indirect peer pressure from his social group, CCHS acquaintances who drink and family members. His friends accept that he has a medical condition that prevents him from drinking alcohol and do not pressurize him to join in.

Ryan has a job he enjoys. When applying for the post, he revealed his medical condition only after he was offered the position for fear of discrimination. Travel to work can be problematic and access-to-work schemes have limited funding. CCHS adults consider them ‘normal’ and do not like applying for funding for physically disabled people. CCHS adults are disadvantaged in the workplace and miss career opportunities because it is difficult for them to work away from home temporarily or permanently.

Independent living is a challenge for these young people. When Ryan leaves home, he will live initially close to his family and have overnight carers. The health authority has agreed to fund 9 hours per night, 7 days a week; this is not a flexible arrangement. There is no daytime care provision. If he needs to sleep during the day due to illness or for other reasons, he would either have to move back home temporarily, or have a family member move in. A service dog would provide additional support for Ryan. However, at present these facilities are not available in UK.

To live independently CCHS adults need to take responsibility for their medical condition. However, parents often overprotect their child and manage their condition for them. For example, Ryan has limited knowledge of how his medical equipment works and his full medical history. At medical appointments, I speak, with his permission, on his behalf. Parents are afraid that their children cannot look after themselves or may take decision that put their lives at risk.

Other issues of independent living include looking after their finances, their household and making decisions about having children. Ryan has a 50% chance of transmitting CCHS to his offspring. He hopes to have children and says he can cope with a child with CCHS. This is not the case for all CCHS adults. Some do not want children for fear of transmitting the condition to their offspring.

Ryan acknowledges that as his parents get older, other support networks will be required. Where will this support come from? His older brother does not live near-by, and it raises the question whether siblings have an obligation to each other?

Ryan does lead a full and happy life, despite all its frustrations. Medical technology is only a small part of his life - other technology, as in the X-box, is a major part of his life! He has a job he loves and an active social life which causes us, his parents, a great deal of stress, but we wouldn’t have it any other way.

### The impact of CCHS on families

***Francesco MORANDI****, MD. Ospedale Sacra Famiglia, Erba , Italy*

Infants and children need nurturing and stable relationships to enable them to develop and grow. These growth- promoting activities are provided by a child’s continuous “serve and return” interaction with a parent or caregiver. They provide experiences that are unique to that child, and stimulate its development [87]. It starts in the first hours of the newborn’s life, when a child is placed in the mother’s arms, and continues through to adulthood. In the past, this cooperative nurturing was performed primarily by the mother within a ‘traditional family’ group with a father and the child’s siblings. Today, effective care is also provided in other social contexts, with around 20% of infants now raised by single parents or within same sex partnerships [88].

The care provided for a child in the first year of life is particularly important. At this time, there is rapid formation and development of neural connections within the brain that will subsequently impact the child’s learning capabilities, adaptive behaviour and life-long physical and mental health. These development changes are influenced not only by the genetic predisposition of the child, but also by the child’s personal and social experiences, and its early physical environment [89].

In the first year of life, a child with CCHS may encounter an unfavourable physical environment and stressful relationships with family and carers. The child may experience severe pain and spend considerable time in intensive care without parental contact. These traumatic circumstances may disrupt the child’s physical and mental development if appropriate support and nurturing is lacking.

The family of a child with CCHS also experiences emotional upheaval. The parents of a 6 month old child with CCHS found the situation overwhelming saying: “We (as a family) have ceased to live.” Stress in these circumstances affects individuals differently. A Parenting Stress Index questionnaire completed by 16 couples, each with a child with CCHS, revealed that the level of stress was near to the risk threshold for the mother but not for the father [90].

Parents’ distress may be compounded by the reactions of those around them. To illustrate this, one parent reported “I saw the panic in the eyes of the health care professionals when my child was diagnosed with CCHS.” They then had to endure their son being labelled by society as ‘that child with Ondine’s curse’ [90].

As parents adapt and reorganise their lives to a child with CCHS, their reactions may evolve through some, or all, of these phases: initial shock, then denial, sadness and anger, before accepting their situation. This process of adaptation is illustrated in the following quotes from parents: “If I had known before how difficult, demanding, time-consuming and exhausting it would be to have my child on a ventilator, I would have never agreed to bring her home”. Over time the parents were proud of her achievement, “Today, after 10 years, we are proud of our Maria despite some sleepless nights, it is a joy for us to see her run, swim, jump, play football (her favourite sport), just like her peers“ [90]. By understanding parents’ reactions, healthcare professionals are better equipped to respond to parents’ emotional needs and provide appropriate support [91].

The impact of CCHS on families and caregivers has not been widely studied. Carers are reported to have low levels of psychological stress, to be highly motivated and to cope well with the demands of looking after a child with CCHS [65,92]. They are also able to safely use all forms of invasive and non-invasive respiratory devices in the home [93]. However, the intensive care and attention the child requires may lead to a high level of marital discord [65,94] and, to mothers having little time to look after their own health [95].

Support groups can help parents and children cope with their unique problems [96,97]. Using the WHO's International Classification of Functioning, Disability and Health, Children and Youth version (ICF-CY), trained health professionals undertook telephone interviews with one parent of each of 26 CCHS children, aged 1.5-17.5 years. The results showed that support by the immediate family was the most important factor in facilitating the development of the child; while that provided by the extended family, healthcare professionals and social services had an ambiguous effect, sometimes facilitating, at other times inhibiting the development of the child. Healthcare professionals should be more responsive to the needs of these families and tailor developmental tasks to the functional abilities of the child [96]. This individualised case management approach has been used to deliver effective clinical care pathways in other chronic conditions, and should be adopted in CCHS [98]. Rare diseases such as CCHS have considerable implications for society. The cumulative burden of rare diseases in the EU is significant, affecting as many as 30 million people. Many parents have no choice but to become full-time carers [99].

The true cost of CCHS to families is not known. A comprehensive epidemiologic survey of 196 patients with a CCHS child has revealed that the disease causes significant financial and psychosocial burden for these families [9]. However, the care which is unpaid is also considerable, life-long and largely unrecognised or acknowledged by society, and as a result rarely evaluated in financial terms [88,100].

More research is required to understand the impact of CCHS on the family and the support required from health and social services. Here the EU consortium may have an important role. Training programmes in the medical humanities should be integrated into medical teaching in universities. This will lead to a more holistic approach to patient care. Hospital routines and clinical pathways should be family-centred, and interventions aimed at improving psychosocial outcomes for these critically- ill children. Programmes, such as NIDCAP (Newborn Individualized Developmental Care and Assessment Program), MITP (Mother-Infant Transaction Programme), COPE (Creating Opportunities for Parent Empowerment) and others, should be actively encouraged as they help improve families’ quality of life and assist parents in developing the lives of their children.

### Future goals in the management of CCHS

***Giancarlo Ottonello.****via Gerolamo Gaslini, Genova, Italy*

CCHS is a rare disorder characterized by a defective response of the autonomic nervous system to hypoxia and hypercapnia. Data from a French epidemiological study has confirmed that CCHS is associated with mutations in the *PHOX2B* gene [13]. Currently, there are no treatments that alter the natural course of the disease. In other rare genetic disorders, such Haemophilia B and Cystic Fibrosis, promising treatment modalities have emerged from an understanding of the underlying genetic basis for these conditions [101,102].

In CCHS, there is progress in identifying candidate drug treatments that modulate the expression of the PHOX2B gene. Several in vitro studies have investigated drugs that promote the clearance of mutant proteins which result from the expression of the *PHOX2B* gene with an expanded polyalanine region [32,103-105]. Animal studies are now required to screen these compounds before clinical trials in CCHS patients. A recent clinical observation has documented a positive response in CCHS patients taking the progestin, desogestrel [106]. This has prompted a clinical study of the efficacy of desogestrel in CCHS patients and the results are expected in the next 24 months [107].

Recent studies on the genetic transmission of the CCHS have identified PHOX2B mutant alleles that are transmitted to offspring from asymptomatic parents. Around 10% of these unaffected parents show somatic mosaicism for the alanine expansion mutation seen in their children [40]. As asymptomatic mutation carriers are at risk of developing alveolar hyperventilation, and bearing a child with CCHS, genetic screening may now be appropriate in these individuals [108].

Although diagnosis of CCHS has improved [13] in some countries there is no consensus on the most appropriate criteria for diagnosis and management of the disease [109]. Earlier diagnosis, together with access to a respiratory ventilator, is required to prevent neurocognitive damage and fatal events in these children. Clinicians need more training to recognise CCHS and tools developed to assist with early diagnosis. Sharing information through the dissemination of case reports [110,111] and close collaboration and development of a centralised database on respiratory diseases of childhood, may lead to a better insight into these conditions [112]. A recent comprehensive epidemiological study, which surveyed the medical and psycho-social complexities of CCHS, found significant deficiencies in the routine evaluation and management of CCHS children [92]. This points to the urgent need for the implementation of guidelines and statements [10,113].

More work is needed to expand our knowledge on the neurological development of CCHS patients. Few papers address this aspect of the disease. A study of the neurocognitive functioning in school-aged children suggests that CCHS may confer risk for adverse neurocognitive outcome [114]. Children and adolescents with CCHS may also have functional and social difficulties in coping with the activities of every day life, which may lead to learning difficulties at school [96]. Healthcare, community and home support is therefore required to improve and nurture the social and intellectual development of these children.

Mechanical ventilators are pivotal to the survival of most children with CCHS. There is a pressing need for healthcare professionals to improve their knowledge of the technical performance of current intensive care and non-invasive home mechanical ventilators. Trigger delay and work load, pressurisation capabilities and cycling profile all impact the performance of these devices. Healthcare staff and patients need to be instructed, on the different characteristics of ventilators and, the precautions necessary for optimal use [115-117]. Development of devices that enhance patient-ventilator synchrony will also help to improve the respiratory assistance required by patients.

Nasal masks for non-invasive, long-term positive pressure ventilation need to be adapted or changed as the child grows to avoid discomfort, skin injury and, to accommodate structural development of the face [118]. Angle Class III malocclusion and severe maxillary hypoplasia treated using devices such as Frankel III orthodontic method [119] and Delaire masks [120] or by LeFort surgery [121] require protocols that standardise procedures to improve outcomes.

Diaphragmatic pacing of the phrenic nerve or of the diaphragm muscle, can improve ventilation and eliminate the need for continuous positive pressure ventilation support. The safety of direct diaphragm stimulation has been demonstrated in a number of respiratory conditions, including a patient with CCHS [77,122]. Obstructive sleep apnoea, arising from phrenic pacing, can be treated by hypoglossal nerve stimulation or a mandible advancement splint [123,124]. This will improve the child’s quality of sleep and as a consequence, improve neuro-behavioural function [125].

CCHS is a condition which requires a multidisciplinary approach at both hospital and community levels. Within hospitals, patients need care from birth, and coordinated care across multiple hospital departments: cardiac, oncology, radiology, endocrinology, neurology and surgical units. Laboratory support for genetic testing, and psychosocial counselling services are also required. In addition, specialised diagnostic screening and surgery equipment is required for the treatment of associated conditions, such as Hirschsprung’s disease [126,127].

Home, community and hospital care support must be integrated: patients should have a follow-up program on discharge from hospital, access to emergency care for treatment and, for respiratory devices, as well as, access to rehabilitation units, psychosocial counselling, home support nurses, school nurses, patient associations and self-support groups. Respiratory care is needed to provide the opportunity for carers to plan holidays and social events.

Home care plays a crucial role in the well-being of patients. Technology is likely to play an increasing role. Breathing parameters recorded at home are comparable with those taken in hospital [128]. The future is likely to involve a combination of home monitoring and telemedicine [129]. Helping CCHS adults to live independently of their families, are initiatives currently under investigation by the Italian [130] and French Families Associations [131]. One study has reported a relationship between the depressive state of a carer of ventilator-assisted children and the number of hours a week a nurse is present to provide support [132]. Home care nursing support, in particular night nursing, appears to be important for the health and well-being of care givers of children with CCHS. However, this support is unlikely to be universally available. Recent research reveals, that even in a developed country, there is a large gap between family members' expectations and provisions of community health care services. Proposals to limit the extremely costly provision of home mechanical ventilation may trigger new ethical dilemmas that should be studied [133].

While there is progress in understanding the genetic basis of the disease, there remain significant challenges in correlating the genotype-phenotype characteristic of the disease, developing effective treatments, co-ordinating care across multidisciplinary healthcare teams and improving ventilator equipment for these patients. Further genetic studies are required to improve diagnosis, characterise the disease and its outcomes.

## Endnotes

^a^HeLa cells are a cell type in an immortal cell line used in scientific research, named after the patient’s name Henrietta Lacks.

^b^The recruitment for this open-label, non-randomized and prospective study ended in September 2013. Of the expected 12, only five patients were included. Only very preliminary results are currently available. The ventilatory response to CO_2_ did not recover in three patients who received desogestrel for the first time in their life. This response deteriorated slightly in two other patients who were previously treated with desogestrel or levonorgestrel when they stopped taking these drugs for the purpose of the study. At this stage, no simple conclusion can be drawn and further analyses are needed. A research program is currently conducted on animal models to better understand the effect of progestin and desogestrel on the control of breathing. It is of highest importance to recall that desogestrel and progestin should not be prescribed to CCHS patients with the goal of restoring their ventilatory response to CO_2_.

## Abbreviations

Alk: Anaplastic lymphoma kinase
ANN: ARtificial neural network
AR: Androgen receptor
ANS: Autonomic Nervous System
bHLH: basic-helix-loop-helix
CB: Carotid body
CCHS: Congenital central hypoventilation syndrome
CHS: Central Hypoventilation Syndrome
EAHC: European Agency for Health and Consumers
Egr2: Early Growth Response Protein 2
EUCHS: European Central Hypoventilation Syndrome
FRAP: Fluorescence recovery after photo-bleaching
HSCR: Hirschsprung disease
ICU: Intensive care unit
Lbx1: Ladybird homeobox 1
LO-CHS: Later-onset CCHS
NI-HMPPV: Non-invasive home mechanical positive-pressure ventilator
nTS: nucleus of the solitary tract
PARM: Polyalanine repeat expansion mutation
PHEA: Public Health European Agency
REM: Rapid eye movement
RTN: Retro-trapezoid nucleus
HMV: Home mechanical ventilation
SSS: Sinus node dysfunction (or sick sinus syndrome)
